# Liquid biopsy for early detection of hepatocellular carcinoma

**DOI:** 10.3389/fmed.2023.1218705

**Published:** 2023-09-22

**Authors:** Ioana Manea, Razvan Iacob, Speranta Iacob, Razvan Cerban, Simona Dima, Gabriel Oniscu, Irinel Popescu, Liliana Gheorghe

**Affiliations:** ^1^“Carol Davila” University of Medicine and Pharmacy, Bucharest, Romania; ^2^Digestive Diseases and Liver Transplantation Center, Fundeni Clinical Institute, Bucharest, Romania; ^3^Center of Excellence in Translational Medicine, Fundeni Clinical Institute, Bucharest, Romania; ^4^Transplant Division, Department of Clinical Science, Intervention and Technology, Karolinska Institute, Stockholm, Sweden

**Keywords:** hepatocellular carcinoma, early diagnosis, liquid biopsy, cell free DNA, extracellular vesicles

## Abstract

Hepatocellular carcinoma (HCC) is a highly prevalent and lethal cancer globally. Over 90% of HCC cases arise in the context of liver cirrhosis, and the severity of the underlying liver disease or advanced tumor stage at diagnosis significantly limits treatment options. Early diagnosis is crucial, and all guidelines stress the importance of screening protocols for HCC early detection as a public health objective. As serum biomarkers are not optimal for early diagnosis, liquid biopsy has emerged as a promising tool for diagnosis, prognostication, and patients’ stratification for personalized therapy in various solid tumors, including HCC. While circulating tumor cells (CTCs) are better suited for personalized therapy and prognosis, cell-free DNA (cfDNA) and extracellular vesicle-based technologies show potential for early diagnosis, HCC screening, and surveillance protocols. Evaluating the added value of liquid biopsy genetic and epigenetic biomarkers for HCC screening is a key goal in translational research. Somatic mutations commonly found in HCC can be investigated in cfDNA and plasma exosomes as genetic biomarkers. Unique methylation patterns in cfDNA or cfDNA fragmentome features have been suggested as innovative tools for early HCC detection. Likewise, extracellular vesicle cargo biomarkers such as miRNAs and long non-coding RNAs may serve as potential biomarkers for early HCC detection. This review will explore recent findings on the utility of liquid biopsy for early HCC diagnosis. Combining liquid biopsy methods with traditional serological biomarkers could improve the overall diagnostic accuracy for early HCC detection.

## 1. Hepatocellular carcinoma worldwide disease burden and current screening strategies

Hepatocellular carcinoma (HCC) ranks as the fifth most common cancer globally in 2023, and the third leading cause of cancer-related death. For males, it is the second, and for females, the sixth cause of cancer-related death (1). Although chronic viral hepatitis incidence is decreasing due to vaccinations and treatments, non-viral factors like metabolic syndrome, NAFLD, obesity, type II diabetes, and alcohol also significantly contribute to liver cancer burden (2). HCC incidence increased by 75% between 1990 and 2015 due to demographic changes, particularly in high socioeconomic index countries (3). In the USA, HCC has tripled in the last 30 years, with specific projections for California showing incidence increases of 318% for Hispanics and 58% for Asians aged 65 and older, between 2014 and 2030 (4).

HCC commonly occurs in the context of liver cirrhosis, and late-stage diagnosis limits treatment options (5). Early diagnosis is vital, with guidelines emphasizing screening protocols for adult patients at-risk, including cirrhotic patients, non-cirrhotic HBV patients at specific risk levels, and advanced fibrosis patients (F3) (6).

Treatment for HCC follows the Modified BCLC staging system. Very early and early stages may lead to curative therapies, like liver resection, ablation, or transplantation. However, only 5–10% of Western patients are diagnosed at these stages, compared to over 30% in Japan (7). For early-stage HCC (BCLC stage A), 5-year survival rates range from 50 to 70% with appropriate treatment (8).

Screening and surveillance employ serological markers and imaging tools. Abdominal ultrasound is widely used, with sensitivity and specificity rates of 57–89% and up to 90%, respectively, (9), but sensitivity drops to 63% for early-stage HCC (10). Recommendations include biannual ultrasounds performed by trained personnel, particularly for liver transplantation candidates.

Early HCC detection via ultrasound is challenging due to cirrhotic liver echotexture and requires skilled personnel and high-quality equipment. The use of contrast agents has not significantly improved the diagnostic capabilities for small HCC lesions (11). Multidetector CT or MRI are not typically used for screening, as they are not cost-effective and are currently reserved for situations where ultrasound examination is inadequate due to obesity, intestinal gas, or chest deformities, or to confirm the diagnosis of HCC.

## 2. Serum biomarkers for HCC screening

Tumor biomarkers are being evaluated for early detection of HCC but have variable sensitivity and specificity. Common biomarkers like AFP, AFP-L3, and DCP are suboptimal in terms of cost-effectiveness for early detection and routine surveillance (6). AFP is extensively used in clinical practice, but variations in levels are also linked to liver regeneration and inflammation. Only a minority of early HCC tumors (10–20%) have elevated AFP (12, 13). Together with ultrasound, AFP increases the detection rate of HCC by only 6–8% (14).

A statistical model called “GALAD” was created using gender, age, AFP-L3, AFP, and des-carboxy-prothrombin, achieving high accuracy for HCC detection regardless of disease stage (15). The GALAD score shows promise for HCC detection but needs further validation in various clinical settings (16, 17). It has been shown that other biomarkers like DCP levels and AFP-L3 are highly predictive for advanced HCC stages, rather than for early detection (18, 19).

Additional serum biomarkers studied for HCC include glypican-3 (GPC3), osteopontin (OPN), and dickkopf-1 (DKK1). GPC3 is a specific biomarker for HCC diagnosis, with several studies focusing on GPC3 serum levels (20–24). Gu et al. developed an effective HCC predictor using MRI-based radiomics signature involving GPC3 with outstanding predictive performance, but the cost-effectiveness for early detection requires further validation (25).

OPN, a glycoprotein over-expressed in various tumors, has been studied for its potential as an additional HCC biomarker to AFP. Salem et al. documented elevated OPN levels in HCC patients, with 73% sensitivity and 54% specificity as a diagnostic marker (26). Duarte-Salles et al. indicated a positive correlation between OPN levels and HCC risk, with improved prediction combining OPN with liver function tests and AFP (27).

Elevated serum level of DKK1 have also been detected in HCC patients, and have been shown to promote inflammation, migration, and invasion through the TGF-β1 signaling pathway (28). DKK1 was proposed as a potential diagnostic biomarker for HCC, but there is still conflicting evidence regarding its role as a promoter or suppressor of metastasis (28, 29). More research is needed to validate the utility of DKK1 for early detection of HCC.

These findings emphasize the ongoing effort to optimize early HCC detection via serum biomarkers but reveal the need for extensive validation and integration into clinical practice as well as for the need of novel biomarkers, with better performance in the HCC screening setting.

## 3. Liquid biopsy for HCC early detection

Recently, liquid biopsy has been proposed as a new tool for diagnosis, prognosis estimation, and patients’ stratification for personalized therapy, in different solid tumors (30). Liquid biopsy uses liquid biological samples such as blood, ascitic fluid or urine, to evaluate useful biomarkers. Circulating tumor cells, circulating tumor DNA (ctDNA), extracellular vesicles (exosomes), or tumor-educated platelets (TEPS) provide novel biomarkers that could be evaluated in liquid biopsies. Plasma is one of the most frequently used biological fluids for liquid biopsy, as it is obtained easily, could be sampled sequentially, at different time points, and, with the use of new genomic and proteomic molecular biology arsenal, could provide robust biological data for various applications, from early diagnosis to personalized therapy. Over the last 10 years, liquid biopsy technologies have significantly advanced, particularly with the introduction of next-generation sequencing.

### 3.1. Cell free DNA for early diagnosis of HCC

Cell-free DNA (cfDNA) analysis offers a noninvasive, real-time liquid biopsy method that accurately represents tumor burden and the genetic profile of HCC. An excellent recent review has focused on presenting the current methods to analyze cfDNA features, current available technical platforms, potential clinical applications including early detection of HCC, as well as current limitations for clinical use (31).

Cell-free DNA are small fragments of degraded DNA (< 200 bp) that originate from disrupted cells and circulate in the bloodstream. cfDNA of malignant origin is called ctDNA. Several recent studies have proposed ctDNA as a better tool for early diagnosis in HCC in comparison to traditional serum biomarkers. The methylation pattern of ctDNA has been investigated in HCC liquid biopsy, as it is a known fact that methylation changes in ctDNA occur early in tumorigenesis (32). It has also been shown that methylation patterns are unique to each cell type and stable under different physiologic or pathologic conditions (33). Furthermore, quantification of cfDNA, assessment of the DNA integrity or the characterization of somatic mutations load, could be valuable tools for detection of HCC, possibly in conjunction with other traditional serological biomarkers. Table 1 summarizes the most significant papers concerning the use cfDNA in liquid biopsies for HCC diagnosis, in studies grouping over 9,000 HCC cases and controls.

**Table 1:** Most significant studies concerning the utility of cfDNA in HCC diagnosis emphasizing percentage of patients in early stages of disease, methodology used to identify ctDNA modification as well as the specific molecular targets.

References	No patients	Main liver disease ethiology	Early Stage	Methodology to identify ctDNA modification	Specific targets
Nguyen et al. (34)	217	HBV	69.1% Clinical Stage I-II	Fragmentomic features of ctDNA, machine learning	12 HCC genes + TERT-promoter region
Guo et al. (35)	596	HBV/HCV	68.4% Clinical Stage I-II	Enzymatic methyl sequencing (EM-seq), NGS	283 HCC specific CpGs out of 1,595 CpGs
Phan et al. (36)	299	HBV	68.9% Clinical Stage I-II	Circulating DNA methylation profiles, NGS	A panel of 450 target regions consisting of 18,000 CpG sites
Kumar et al. (37)	230	HBV	38% BCLC A	RT PCR, integrity index determined by the absolute quantitation method	Repetitive elements (ALU and LINE1) and housekeeping genes (β-Actin and GAPDH)
Wang et al. (38)	747	HBV	Training set case–control study; Validation in HBV asymptomatic carriers cohort, during HCC screening	NGS, Multiplex profiling of cfDNA, simultaneous detection of genetic and epigenetic alterations	Mutation Capsule Plus (MCP) Technology
Foda et al. (39)	724	HCV	32% BCLC 0-A	Genome-wide cfDNA Fragmentation Profiles by Low-coverage WGS (2.6x)	473 nonoverlapping 5-Mb regions
Sun et al. (40)	452	HBV	35.1% BCLC 0-A	Genome-wide cfDNA Fragmentation Profiles by Low-coverage WGS (1.49× to 4.65×)	472 nonoverlapping 5-Mb regions
Kim et al. (41)	433	N/A	89.4% Stage T I-II	High-resolution melting (MS-HRM) analysis	methylation levels of the USP44 promoter
Lee et al. (42)	249	HBV	71.8% Single tumor	ddPCR	AFP
Jiao et al. (43)	119	N/A	33.3% Clinical Stage I-II	Targeted NGS, HiSeq4000 Sequencer Illumina	T200.1 panel that covers 262 cancer-associated genes
Kunadirek et al. (44)	30	Non-viral	36.6% BCLC 0-A	Whole exome sequencing	ZNF814, ZNF492, and ADAMTS12
Wang et al. (45)	223	N/A	15.4% BCLC A	DNA methylation quantification using a ddPCR platform	cfDNA methylation ratio
Chen et al. (46)	3,204	HBV	41.8% BCLC 0-A	Next generation sequencing	Genomewide 5-hydroxymethylcytosine (5-hmc), nucleosome footprint (NF), 5′ end motif4 and fragmentation profiles of cfDNAs
Cai et al. (47)	362	HBV	N/A	Genome-wide 5hmC sequencing of cfDNA samples	64 5hmC signatures in cfDNA
Kisiel et al. (48)	244	HBV	48% BCLC 0-A	TELQAS assays (NGS)	6 methylation markers
Ng et al. (49)	30	HCV	67% BCLC A	NGS Ion S5XL	46 gene panel
Oussalah et al. (50)	289	HCV	26% BCLC A	Methylation MS-PCR	SEPT 9
Wu et al. (51)	494	HBV/ HCV/ alcohol	100% of cases from population-based screening	Methylation, Pyrosequencing, rtPCR	TBX2
Lu et al. (52)	180	HBV	79.4% Clinical Stage I-II	Methylation MSPCR	APC, COX2, RASSF1A
Huang et al. (53)	48	HBV	98% BCLC A	ddPCR	TERT, TP53, CTNNB2
Liao et al. (54)	51	HBV	73% Single tumor	NGS (Myseq)	TERT, TP53, CTNNB1
Jiang et al. (55)	225	HBV	94% BCLC A	HiSeq2000 NGS	Chromosome arm-level z-score analysis (CAZA)
Huang et al. (56)	109	HCV	26% TNM I	Pyrosequencing, Methylation, CpG p16 Kit, PyroMark Q24	P16
Han et al. (57)	293	HBV	58.7% TNM I-II	Methylation MS-PCR	TRG5
Ji et al. (58)	189	HBV	53% TMN I-II	Methylation MS-PCR	MT1M, MT1G
Piciocchi et al. (59)	142	HCV	59% within Milan	qRT-PCR	TERT
Sun et al. (60)	93	HBV	58% TMN I-II	Methylation MS-PCR	TFPI2
Yang et al. (61)	110	HBV	45% TMN I-II	Real-time quantitative fluorescent PCR	TERT
Iizuka et al. (62)	422	HCV	52% single lesion	Methylation MS-PCR	SPINT2, SRD5A2
Tangkijvanich et al. (63)	208	HBV	48% CLIP<2	Methylation. PCR - Combined bisulfite restriction analysis	LINE-1 repetitive sequences

#### 3.1.1. Methylation pattern in cfDNA

Epigenetics plays a crucial role in oncologic disease development, with methylation analysis of circulating cell-free DNA (cfDNA) offering significant information. Pre-analytical variables, including cfDNA extraction and bisulfite conversion methods, impact methylation identification. Despite advancements in simplifying methylation analysis, it remains a complex procedure, and a standardized pre-analytical process for methylated circulating DNA is lacking. In this setting, Alain Thierry’s group has recently published a comprehensive review of pre-analytical, analytical, and patient-related factors proposing guidelines for analyzing methylated circulating DNA in liquid biopsies (64).

Several recent papers have focused on the utility of methylation profiles for HCC screening. Guo et al. recently demonstrated that epigenetic variants in ctDNA can serve as early HCC detection biomarkers. Enzymatic methyl sequencing (EM-seq) revealed that enzymatic conversion of unmethylated C to U outperformed bisulfite conversion. The researchers identified 283 CpGs with significant methylation differences between HCC and non-HCC samples. A screening model based on these markers effectively distinguished HCC samples with an area under the curve of 0.957, performing well across various stages independent of the serum AFP/PIVKA-II status (35). Phan et al. utilized HCC-specific circulating DNA methylation profiles to enhance the accuracy of existing screening assays for patients at high-risk to develop HCC. By identifying differentially methylated regions in cfDNA between HCC and high-risk controls with liver cirrhosis or chronic hepatitis, they trained machine learning classifiers. The model differentiated HCC from high-risk non-HCC patients with an area under the curve of 0.84. Combining these markers with three traditional serum biomarkers in a commercial test, achieved an area under the curve of 0.87, with 68.8% sensitivity and 95.8% specificity to correctly identify HCC. This study suggests that adding cfDNA methylation signatures to serological biomarkers could improve liver cancer detection accuracy (36). The SEPT9 gene, a crucial cell division regulator and tumor suppressor, is linked to liver carcinogenesis through hypermethylation. Oussalah et al. assessed a PCR-based assay’s diagnostic accuracy for analyzing SEPT9 promoter methylation in circulating cell-free DNA (mSEPT9) among cirrhotic patients, for HCC diagnosis. The mSEPT9 test displayed high diagnostic accuracy, with AUROCs of 0.944 and 0.930 in the training and replication cohorts, respectively. The authors suggested that the mSEPT9 test could be a promising circulating epigenetic biomarker for HCC diagnosis in cirrhosis patients, potentially benefiting screening protocols (50).

Other groups have shown that detection of concurrent plasma or serum p15 and p16 methylation was positive in 92% of HCC at diagnosis, whereas RASSF1A promoter hypermethylation was detected in 90% of HCC, differentiating HCC cases from chronic HCV infections and healthy controls (65, 66). Similarly, HCC could be differentiated from normal controls with good sensitivity and specificity by quantitative analysis of multiple methylated genes in plasma (APC, GSTP1, RASSF1A, and SFRP1) (67). By adding miRNA information to the methylation profile of APC, COX2, RASSF1A, a predictive model to diagnose HCC in patients with low AFP values was generated (52).

#### 3.1.2. cfDNA somatic mutations for HCC detection

cfDNA is an attractive source of genomic information with interesting considerations concerning potential circumventing intratumoral heterogeneity. Quantification of cfDNA together with detection of defined somatic mutations could be used to facilitate early diagnosis in HCC (68). De Mattos-Arruda et al.’s proof-of-principle study demonstrated that using high-depth targeted massively parallel sequencing of plasma-derived ctDNA could identify tumoral somatic mutation burden and could be used for monitoring somatic genetic alterations, addressing challenges presented by intra-tumor genetic heterogeneity (69). Huang et al. aimed to minimize the impact of intratumoral heterogeneity on profiling hepatocellular carcinoma by using whole exome sequencing (WES) and targeted deep sequencing (TDS) of multi-regional tumor samples and paired cfDNA. They found that heterogeneity decreased at higher TDS sequencing depth compared to WES. Despite increased cfDNA genome profiling efficiency with sequencing depth, an average of 47.2% total mutations were detected by blood mononuclear cells under TDS, suggesting that tissue outperforms cfDNA, with the latter potentially serving only as an alternative for profiling HCC genomes (53). On the other hand, the sensitivity of the method used for plasma cfDNA profiling is a critical issue, and in this setting the best results have been achieved using droplet digital PCR (69, 70).

A recent study focused on assessing the diagnostic and prognostic value for HCC of a new panel of somatic mutations in liquid biopsy. Concordance of tissue and plasma cfDNA ranged from 52 to 84%, whereas for selected mutations AUROC for HCC diagnosis was 0.92 and increased to 0.96 in combination with AFP (71).

Among the most frequent somatic mutations in HCC are TERT promoter mutations C228T and C250T, encountered in up to 50% of HCC. However, they have also been identified in cirrhosis, as a premalignant state (72). Although considered as a single biomarker, TERT promoter mutations do not seem ideal for HCC screening, the detection of these mutations in liquid biopsy, in conjunction with other genetic or epigenetic biomarkers, could define better the premalignant state, indicating a subgroup of patients in which aggressive surveillance for HCC is mandatory.

According to the COSMIC database, together with TERT promoter mutations, somatic variants in TP53 (rs28934571) and CTNNB1 (rs121913412 and rs121913407) are frequently encountered in HCC and could be of interest for HCC screening and surveillance. In contrast to TERT promoter mutations, they are more specific to the malignant phenotype. In an earlier study of Huang et al., TP53, CTNNB1, and TERT (c.1-124C > T) were investigated by ddPCR in HCC, with promising results. At least one of the circulating mutants was identified in 56.3% of patients, with the mutant allele frequency ranging from 0.33 to 23.7% (53). Lee et al. employed a highly sensitive cfDNA detection system combined with machine learning to enhance HCC detection and prognosis. Among several serum/plasma biomarkers, alpha-fetoprotein (AFP) expression in captured cfDNA showed the highest accuracy for diagnosing malignancies. By developing a cfDNA score, which integrated total plasma cfDNA levels and cfAFP-DNA expression using machine learning algorithms, the diagnostic/prognostic capability of cfDNA was further improved. ROC curve analysis revealed that the cfDHCC score could better differentiate HCC patients based on tumor UICC stage, detect multifocal tumors, and estimate tumor sizes compared to plasma cfDNA or cfAFP-DNA alone (42). Kunadirek et al. demonstrated the value of whole-exome sequencing (WES) of cfDNA for determining somatic mutation profiles of HCC in Thai patients. CfDNA levels were significantly higher in HCC patients compared to chronic hepatitis patients. Somatic SNP variants were identified in cfDNA, including ZNF814 (27%), HRNR (20%), TP53 (17%), ADAMTS12 (17%), and TTN (17%). These mutations were also identified in data from The Cancer Genome Atlas (TCGA) from a previous study in Thai population, supporting cfDNA as a reliable biomarker for HCC detection (44).

In a recent meta-analysis, Li et al. grouped the most important studies published up to January 5th 2022, examining the diagnostic performance of ctDNA as a minimally invasive biomarker for HCC. They investigated different subgroups of studies: qualitative or quantitative ctDNA analyses, combined alpha-fetoprotein (AFP) and ctDNA assay, and the diagnostic value of methylated SEPTIN9 (mSEPT9). A total of 59 articles with 9,766 subjects were included in the final analysis. Qualitative studies had an integrated sensitivity of 0.50, specificity of 0.78, and AUC of 0.78. Quantitative studies yielded sensitivity, specificity, and AUC values of 0.69, 0.84, and 0.81, respectively. Six studies evaluating mSEPT9 had an AUC of 0.86, sensitivity of 0.80, and specificity of 0.77. Combining AFP with ctDNA assay resulted in an AUROC of 0.89, sensitivity of 0.82, and specificity of 0.84. The study demonstrated that ctDNA, especially mSEPT9, holds good diagnostic potential in HCC, but combining ctDNA with conventional serological assays like AFP can enhance diagnostic performance (73).

#### 3.1.3. Cfdna fragmentome features to detect HCC

Besides methylation and mutations profiles, cfDNA fragment features like size, jagged ends, and endpoint locations have been utilized to create noninvasive cancer screening and diagnostic assays (74). A study on patients with HBV, cirrhosis and healthy controls found that the majority of cfDNA fragments had a median length of 166 bp (close to the size of a nucleosome). However, the distribution of fragment lengths in a patient’s plasma correlated with the amount of ctDNA. Patients with a higher percentage of ctDNA had a slight deviation toward smaller fragments (<150 bp) and patients with lower ctDNA percentage had a slightly higher number of large fragments (>180 bp), while the median stayed at 166 bp (55). Cancer patients’ cfDNA revealed multiple genomic differences, including longer and shorter fragments in different regions. Studies demonstrated that mutant allele-bearing cfDNA fragments were often shorter and selecting for shorter fragments increased ctDNA proportions in samples (75). Prior research indicates cfDNA fragmentation is a non-random event influenced by apoptotic-dependent caspases. Non-tumor cfDNA fragment size distribution displays at average a 167 bp size, corresponding to DNA wrapped around histones (147 bp) and a linker region (10 bp), while ctDNA fragments measure around 145 bp (76). This suggests that ctDNA is represented by the shorter fragments in a cfDNA pool. Size differences arise from variations in nucleosomal organization and chromatin accessibility between non-tumor cfDNA and ctDNA, ctDNA exhibiting a more accessible chromatin than non-tumor DNA, potentially due to the highly active transcriptional state of these regions (77).

The study group led by Professor Velculescu was among the first to analyze cfDNA fragmentation patterns for HCC detection. They previously developed a method known as DELFI (DNA Evaluation of Fragments for Early Interception), which uses genome-wide fragmentation profiles to offer a cost-effective and high-performing solution for cancer detection. The research team from Johns Hopkins recently improved this technique by investigating the molecular origins of cfDNA in HCC patients, identifying genomic and chromatin characteristics linked to fragmentation alterations. This method was employed to detect liver cancer in US patients and validated in a separate Hong Kong cohort, in a total study group of 724 cases and controls. The machine learning model incorporating multifeature fragmentome data, achieved 88% sensitivity and 98% specificity in detecting HCC within an average-risk population, as well as 85% sensitivity and 80% specificity among high-risk individuals (39). The DELFI approach was also evaluated for liver cancer surveillance and detection in a theoretical population of 100,000 high-risk individuals using Monte Carlo simulations, focusing on early-stage cancer detection. Compared to the current standard of ultrasound and AFP, with 39% adherence in the U.S., the DELFI approach assumed 75% testing adherence, as blood tests offer higher accessibility and compliance. The simulations revealed that DELFI would lead to a 2.46-fold increase in liver cancer detection, compared to ultrasound with AFP alone. It was estimated that this would decrease the false-negative rate from 38% (for ultrasound with AFP) to 24% for DELFI and increase the negative predictive value (NPV) from 95.7 to 97.1%. The results suggest significant benefits in using the DELFI approach as a high-specificity, blood-based early detection tool for liver cancer (39).

In a recent study, Nguyen et al. used concurrent analysis of cancer-related mutations and fragment length profiles to discriminate mutation sources. They generated a classification model to distinguish HCC patients from healthy controls using three different ctDNA fragmentomic features, via deep sequencing of 12 HCC genes + the TERT-promoter region. The model achieved an AUROC of 0.88, 89% sensitivity, and 82% specificity in the discovery cohort, while in the validation cohort, achieved an AUROC of 0.86, with 81% for both sensitivity and specificity (34).

Jiang et al. employed massively parallel sequencing for single-base resolution plasma DNA size measurement, in a genome-wide manner. Using chromosome arm-level z-score analysis (CAZA), they identified tumor-derived plasma DNA molecules and examined their size profiles. They found abnormally short and long DNA molecules in HCC patients’ plasma, with short ones carrying tumor-associated copy number aberrations. Elevated levels of shorter mitochondrial DNA were observed in HCC patients’ plasma, compared to healthy subjects. Using fractional concentrations of mitochondrial DNA in plasma, with a cutoff of 0.00084%, an AUROC of 0.93 was achieved, with 80% sensitivity and 94% specificity for distinguishing HCC patients from healthy individuals (55).

In a large cohort of 3,204 cases and controls, Chen et al. used state-of-the-art NGS technology to obtain genomewide profiles for 5-hydroxymethylcytosine (5-hmc), nucleosome footprint (NF), 5′ end motif and fragmentation profiles of cfDNA. They constructed a diagnostic model using logistic regression based on the four features. The integrated HIFI (5-Hydroxymethylcytosine/ motIf/ Fragmentation/ nucleosome footprInt) method demonstrated strong diagnostic value, differentiating HCC from liver cirrhosis with 95.42% sensitivity and 97.83% specificity in the test cohort, and 95.79% sensitivity and 95% specificity in the validation set, respectively. HIFI outperformed AFP in differentiating HCC vs. liver cirrhosis and showed promise in differentiating HCC from non-HCC and normal controls (46).

### 3.2. Extracellular vesicles in liquid biopsies for early diagnosis of HCC

It is now widely accepted that intercellular communication occurs not only through direct contact between cells, for example mediated by tight junctions or proteins or through soluble factors (proteins or lipids) in an autocrine, paracrine, and endocrine scenario, but also through released extracellular vesicles (EVs) (78). Small extracellular vesicles research has made significant advances in the last decade, emphasizing their importance as ctDNA, protein, and non-coding RNA carriers, relevant for early diagnosis, disease prognosis, and personalized therapy applications.

EVs are a heterogeneous group of membranous “cargo” vesicles comprising of proteins, lipids, RNA, DNA and miRNA, packaged within a lipid bilayer. They are released into the extracellular environment from healthy, inflamed, malignant, or dying apoptotic cells (79). EVs are classified based on their biogenesis and size into small EVs (also called exosomes, with sizes <100 nm or < 200 nm) and large EVs (also called microvesicles (MVs), with sizes >200 nm) (80). Exosomes originate from the fusion of multivesicular endosome (MVEs) with the plasma membrane, while microvesicles are shed directly from the plasma membrane via the membrane budding process. With increasing the understanding of the role of these EVs, what was once thought to be a cellular by-product of biological insignificance, is now being explored for its profound clinical utility. EVs cargo composition depends on the pathologic and physiologic state of their cells of origin. They are released into the extracellular environment and can be detected in the serum, plasma, urine, saliva, etc. Moreover, they are protected from degradation by the lipid bilayer, increasing their resistance to RNases, making them an attractive noninvasive liquid biomarker to provide a snapshot of their cells of origin. At present, no unique markers have been identified to differentiate exosomes from other nanoscale vesicles, and this could evidently limit their specific detection in human fluids (81). The liver is a multicellular organ made of parenchymal cells (hepatocytes and cholangiocytes) and non-parenchymal cells (Kupffer cells, hepatic stellate cells, and sinusoidal endothelial cells), each capable of producing different types of EVs, and hence hepatic EVs play a key role in intercellular communication to maintain homeostasis. They could serve as liquid biopsy biomarkers to aid in the diagnosis and prognosis of various liver diseases including alcoholic and non-alcoholic steatohepatitis (NASH), viral or non-viral liver cirrhosis, and HCC (82). A very recent review paper has addressed the multi-omics applications in hepatic precancerous lesions and hepatocellular carcinoma with special focus on extracellular vesicles (83). The main EVs detection methods used in studies were high sensitivity flow cytometry, ELISA, and qRT-PCR. Thus, methods of isolation, detection, and characterization need to be standardized in the future to facilitate comparison. The latest, most relevant, studies published on the role of EVs and their cargo for HCC early diagnosis are summarized in Table 2.

**Table 2:** Most relevant studies published on the role of EVs and their cargo for HCC diagnosis, emphasizing percentage of patients in early stages of disease.

Study	EVs cargo target	Number of Patients,	Early stage HCC	Validated maker used for comparison	Main Findings (Sensitivity/Specificity, AUC)
Rui et al. (84)	miR-425-5p, let-7d-5p, miR-122-5p	124 HCC, 46 non-tumor donors	52.4% Stages I-II	N/A	Training cohort: AUC for HCC miR-425-5p, let-7d-5p, miR-122-5p, 3 miR signature: 0.65, 0.68, 0.83, 0.95. AUC for early HCC: 0.70, 0.76, 0.84, 0.92
Yao et al. (85)	exosomal lncRNA H19-204, THEMIS2-211 and PRKACA-202	168 HCC, 101 normal controls	64.8% TMN I-II	AFP	Combined score (H19-204 + LncRNA THEMIS2-211+ PRKACA-202) had AUC, Se, Sp for HCC in the validation set: 0.88, 70.9, 94.6%; LncRNA THEMIS2-211 for aerly stage HCC had AUC, Se, Sp of 0.81, 82.8, 70.8%
Chen et al. (86)	miR-34a	60 HCC, 60 healthy controls	48.3% TMN I-II	AFP	Sensitivity and specificity of serum exosomes miR-34a, AFP and their combined detection for the diagnosis of HCC were 78.3 and 51.7%, 61.7 and 98.3%, 68.33 and 93.33%, respectively; exo-miR-34a + AFP AUC 0.85.
Guo et al. (87)	exo_circ_0006602	87 HCC, 30 healthy controls	100% early stage	AFP	All Stages HCC, sensitivity 0.77; specificity 0.933; AUC 0.907. In cases of TNM stage I, the AUC value was 0.9564, Se 93.3%, Sp 89.7%
von Felden et al. (88)	3 small RNA clusters signature - smRC (unannotated)	105 HCC (), 85 CLD, 19 healthy controls	100% BCLC 0-A	AFP	3-smRC signature for early stage HCC has an AUC 0.87, 86% sensitivity, 91% specificity, 89% positive predictive value, significantly better than AFP + US.
Kim et al. (89)	onco-lncRNAs, DLEU2, HOTTIP, MALAT1, and SNHG1	72 HCC, 21 chronic hepatitis, 25 liver cirrhosis, 21 healthy controls	100% Very early stage, mUICC I	AFP	For all-stage HCC diagnosis, the combination of AFP and EV-MALAT1 had AUC 0.91 (HCC vs. nontumor); EV-MALAT1 and EV-SNHG1 had AUC 0.88 (HCC vs. CH/LC); For Very early HCC (mUICC stage I) EVDLEU2 and EV-MALAT1, and EV-HOTTIP and EVMALAT1 combinations had best AUC of 0.92 (HCC vs. nontumor) whereas combination EV-MALAT1 and EV-SNHG1 had AUC 0.98 for (HCC vs. CH/LC)
Lyu et al. (90)	hsa_circ_0070396	111 HCC, 50 chronic hepatitis, 58 liver cirrhosis, 54 healthy controls	28% TNM I-II	AFP	AUC, Se, Sp for HCC vs. healthy controls 0.857, 62.16, 98.15%; HCC vs. chronic hepatitis 0.774, 76.58, 68%; HCC vs. liver cirrhosis 0.66, 46.85, 81.03%
Hao et al. (91)	miR-320a	104 HCC; 55 CLD; 50 healthy controls	37.8% TNM I-II	AFP	HCC vs. Healthy controls (Se/Sp, AUC): 77.9%/80%, 0.86. AUC value for serum exosomal miR-320a was 0.860 with a sensitivity; HCC vs. CLD (Se/Sp, AUC): 71.6%/81.8%, 0.83
Cui et al. (92)	LDHC	112 HCC, 100 healthy controls	44.6% Stages I-II	N/A	AUC of exosomal LDHC in distinguishing early-stage HCC patients from healthy controls 0.9451, sensitivity and specificity 88.2 and 93.3%
Wang et al. (93)	miR-122, miR-21, miR-96	50 HCC, 50 LC, 50 healthy controls	20% TNM I	AFP	miRNA panel had high accuracy in discriminating HCC from the cirrhosis group (AUC 0.924; sensitivity 82%, specificity 92%)
Sorop et al. (94)	miR-21-5p, miR-92a-3p	48 HCC (), LC 38, healthy controls 20	87.5% BCLC A	AFP	AUC of combined miR/AFP score for HCC diagnosis 0.85
Cho et al. (95)	miR-25-3p, miR-140-3p, miR-423-3p, miR-1269a, miR-4,661-5p, and miR-4,746-5p	147 HCC, 42 liver cirrhosis, 46 chronic hepatitis, 41 normal controls, in 3 cohorts: screening, test and validation	31.3% Stages I-II	AFP (>20 ng/mL)	mUICC I and II vs. CH and LC (Se/Sp, AUC): miR-4,661-5p + miR-4,746-5p: 86.6%/90.4%, 0.95; mUICC I vs. CH and LC (Se/Sp, AUC): miR-4,661-5p + miR-4,746-5p: 86.6%/93.1%, 0.95
Lu et al. (96)	lncRNAs: ENSG00000248932.1, ENST00000440688.1, ENST00000457302.2	200 HCC (), 200 CLD, 200 healthy controls	49%/34% TNM I/II	AFP	Three lncRNAs: AUC 0.96/0.53 in training/validation cohorts; Three lncRNAs vs. AFP: 0.97/0.87 in training/validation cohorts
Li et al. (97)	lncRNAs	71 HCC, 37 CLD, 94 healthy controls	63% early-stage HCC	AFP (>10 ng/mL)	Support vector machine model (Se/Sp, AUC): Training set: 84%/94%, 0.95; Validation set: 89%/91%, 0.98; Testing set: 85%/95%, 0.96; Among patients with AFP measurements (60 HCC, 17 with benign liver lesions): Support vector machine model: AUC, 0.95; AFP alone: 0.83 (*p* = 0.037)
El Gwad et al. (98)	lncRNA-RP11-513I15.6, miR-1,262 and RAB11A	60 HCC, 42 CLD, 18 healthy controls	90% BCLC 0/A	AFP	All stages HCC Combined (RP11-513I15.6 + miRNA 1,262 + AFP) Se/Sp/PPV/NPV/Accuracy: 100%/76.7%/81.1%/100%/88.3%; Early stage HCC: 100%/76.7%/79.4%/100%/ 87.7%
Pu et al. (99)	miR-144-3p, miR-21-5p	24 HCC; 16 CLD, 17 healthy controls	38% BCLC stage 0	AFP	miR-144-3p: AUC 0.75; miR-21-5p: AUC 0.44; miR-144-3p + miR-21-5p: AUC 0.78; AFP: AUC 0.63 (*p* > 0.05 vs. all miR groups)
Wang et al. (100)	miR-122, miR-148a and miR-1,246	68 HCC; 53 LC; 50 CLD; 64 healthy controls	74% TNM I-II	AFP	Early stage HCC vs. cirrhosis: miR-122: AUC 0.80; miR-148a: AUC 0.86; miR-1,246: AUC 0.76 AFP: AUC 0.67; miR-122 + miR-148a + AFP: AUC 0.95
Xu et al. (101)	hnRNPH1 mRNA	88 HCC; 135 CLD, 68 healthy controls	16% TNM I-II	AFP (>20 ng/mL)	HCC vs. chronic hepatitis: hnRNPH1 alone: 85.2%/76.5%, AUC 0.87; AFP alone: 69.3%/87.9%, AUC 0.79; hnRNPH1 + AFP: AUC 0.89 (*p* < 0.05 vs. AFP); HCC vs. cirrhosis: hnRNPH1 alone: 86.4%/54.0%, AUC 0.65; AFP alone: 46.6%/88.3%, AUC 0.67 hnRNPH1 + AFP: AUC 0.75 (*p* < 0.05 vs. AFP)
Xu et al. (102)	ENSG00000258332.1 and LINC00635 (expression relative to GADPH)	115 HCC; 241 CLD, 120 healthy controls	26% TNM I-II	AFP (>20 ng/mL)	Training cohort: HCC vs. chronic hepatitis (Se/Sp, AUC): ENSG00000258332.1: 71.6%/83.4%, 0.72; LINC00635: 76.2%/77.7%, 0.75; AFP: 54.7%/75.3%, 0.67; All 3 markers: 83.6%/87.7%, 0.89 (*p* < 0.05 vs. AFP); Validation cohort: HCC vs. chronic hepatitis (Se/Sp, AUC): ENSG00000258332.1: 73.5%/80.5%, 0.72; LINC00635: 79.6%/75.2%, AUC, 0.73; AFP: 52.5%/74.1%, 0.63; All 3 markers 84.5%/85.3%, 0.89 (*p* < 0.05 vs. AFP)

It has been shown that the levels of peripheral blood MVs were significantly increased in HCC patients compared with liver cirrhosis, and were correlated to the tumor size, progression, and stage of the disease. The AUROC for microvesicle discriminating patients with TNM stage I and TNM stage II HCC from cirrhosis was found to be 0.83 (95% CI 0.74–0.93) and 0.94 (95% CI 0.88–1.00), respectively (103).

Oxidized mtDNA-enriched EVs and acetaldehyde can jointly trigger oxidative stress and oncogenic pathways in alcohol-related liver cancer (104). NGS analysis revealed EV mtDNA differences between HCC patients and healthy controls concerning mtDNA end sites, cleavage numbers, and copy numbers, suggesting potential diagnostic value (105). Tumor-derived EVs show promise as disease-specific biomarkers; however, conventional isolation techniques struggle to separate tumor-specific EVs. Sun N et al. developed the EV Click Chips system, a covalent chemistry-based solution, for efficient and pure isolation of plasma HCC-derived EVs (106).

Non-coding RNAs including microRNA (miRNA) (18–22 nt long), long non-coding RNA (lncRNA) (>200 nt long) and circular RNAs are now emerging as a potential biomarkers in multiple diseases because of their presence in the body fluids and rapid variation with disease stages. Among the cargos of EVs are miRNAs. Exosomal miRNAs are protected from RNase digestion, which enhances their stability and diagnostic potential as a biomarker.

miRNAs are endogenous, non-coding RNAs involved in post-transcriptional regulation of gene expression, by inducing degradation of mRNA following complementary binding to 3′-untranslated regions of target mRNAs. There were multiple miRNAs identified that have a significant impact on gene expression controlling cellular proliferation, differentiation, or apoptotic pathways. Each miRNA controls multiple gene transcripts, in complex regulatory networks, and are valuable biomarkers for prognosis, targeted therapy as well as early diagnosis of various oncologic conditions. miR21 was found to be a better diagnostic marker than AFP for HCC (107), whereas miR130b had an AUROC of 0.91 for HCC and together with miR15b was proposed as a biomarker for early diagnosis in HCC. Furthermore, seven miRNAs were included in a panel for early detection of HCC occurrence in the setting of HBV chronic infection: miR-122, miR-192, miR-21, miR-223, miR-26a, miR-27a, and miR-801 – AUROC 0.88 (108, 109).

Our study group has recently conducted a comprehensive transcriptomic analysis of tissue, serum, and serum exosomes from hepatocellular carcinoma patients, indicating a correlation in miRNA expression between exosomes, serum, and tissue samples, suggesting export from tumors via exosomes. miR21 was documented as a diagnostic and prognostic biomarker (110). Based on these data, we have further validated two miRNA targets indicated by the above-mentioned small RNA sequencing study, miR-21-5p and miR-92a-3p, as potential biomarkers for hepatocellular carcinoma screening. Our study cohort included 106 patients, 48 diagnosed with HCC during screening protocol, 38 liver cirrhosis patients, on the waiting list for liver transplantation, and 20 healthy volunteers. The exosomal expression level of miR-21-5p and miR-92a-3p were used together with serum AFP to generate a score that could be used for HCC diagnosis. The score achieved an AUROC of 0.88 for HCC diagnosis, better than AFP alone (0.77) (94).

The addition of genetic biomarkers such as somatic mutations frequently encountered in HCC could further refine scores used for early diagnosis of HCC. The use of both genetic and epigenetic biomarkers in liquid biopsy is a novel approach to screening and surveillance for HCC, that holds great promise by emphasizing sensitive tumoral molecular features significant for early diagnosis.

Numerous lncRNAs and circRNAs from EVs have been suggested as potential HCC biomarkers. LncRNAs, non-coding RNAs exceeding 200 nucleotides, regulate the transcriptome and are linked to tumor cell transformation (111). LncRNA expression in HCC patient EVs is significantly higher than in chronic hepatitis/cirrhosis or healthy controls, offering better diagnostic ability than AFP for early-stage HCC. CircRNAs, endogenous non-coding RNAs with a closed-loop structure, are enriched and stable in EVs (112). Hu K et al. observed increased circCMTM3 levels in HCC patient exosomes, which promote angiogenesis via the miR-3,619-5p/SOX9 axis, thus encouraging HCC tumorigenesis (113). Conversely, exosome-delivered circ_0051443 inhibits HCC cell malignancy by inducing apoptosis and cell cycle arrest, with plasma EV expression in HCC patients significantly lower than in healthy controls (AUC: 0.8089) (114). Transcriptome sequencing and large sample clinical validation identified circ_0006602 and circ_0028861 in EVs as HCC candidate biomarkers, suitable for early diagnosis and screening (87).

## 4. Strengths and limitations of using liquid biopsy for early detection of HCC in clinical practice

**Figure 1:**
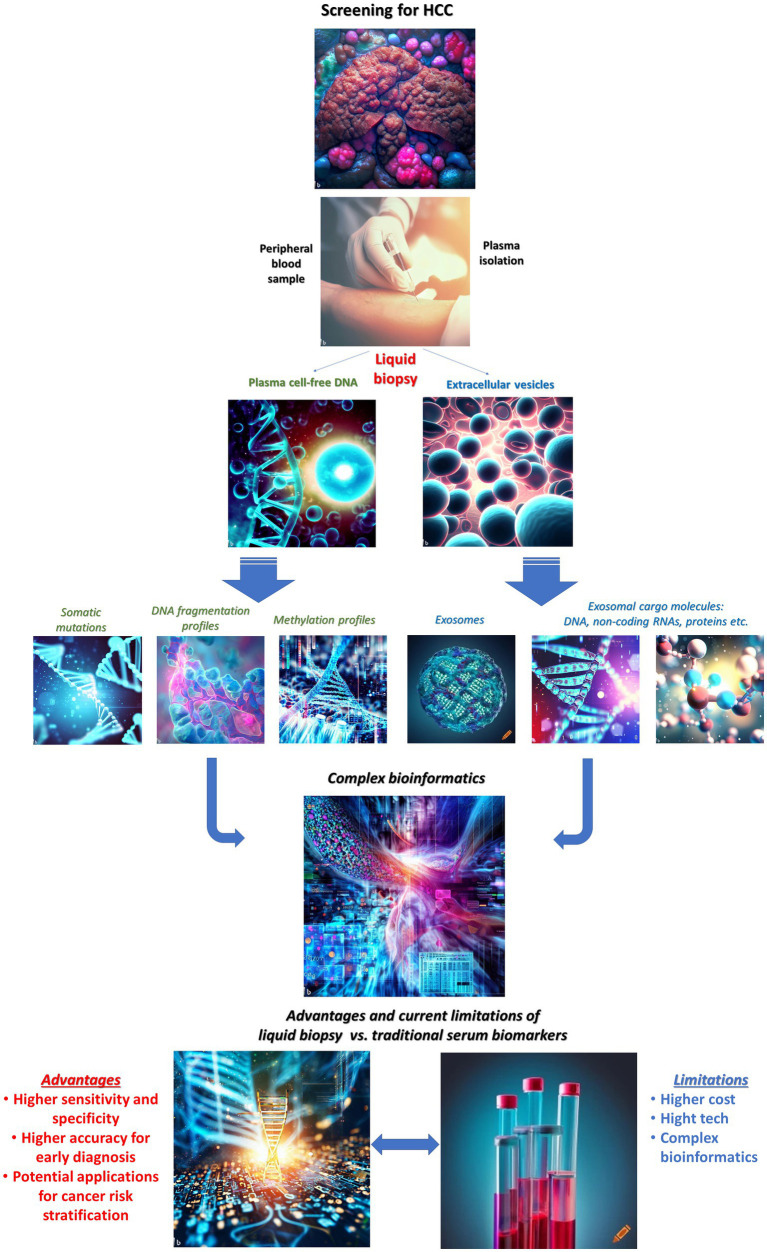
Liquid biopsy for early detection of hepatocellular carcinoma: current state for clinical applications, strengths, and limitations. Images have been generated using AI-powered “text to image” technology, available online: Bing Image Creator and Crayon.

Liquid biopsy is an emerging early diagnosis tool in cancer, including HCC. The present approach to HCC screening for early diagnosis using plasma liquid biopsy, emphasizing strengths, and limitations, in comparison to traditional serum biomarkers is schematically depicted in Figure 1. Whereas some liquid biopsy techniques, such as circulating tumor cells (CTCs), have mainly found a better suitability for personalized therapy and prognosis, cell free DNA and extracellular vesicle-based technologies have been investigated with promising results for early diagnosis, HCC screening and surveillance protocols.

Several cfDNA features can be used in this setting: methylation patterns in cfDNA, cfDNA somatic mutations, cfDNA fragmentome features.

There are several strengths of cfDNA liquid biopsy that could further support this technology in the clinical setting. cfDNA is obtained from blood samples, making the procedure less invasive than tissue biopsies, and ideal for screening procedures. It allows for early diagnosis, detection of recurrence following therapy, real-time monitoring of tumor dynamics, and response to therapy. cfDNA may detect HCC earlier than traditional serological biomarkers, such as alpha-fetoprotein (AFP), due to its higher sensitivity and specificity. Aberrant methylation patterns or specific mutations are characteristic to tumor cells, providing a distinct signature that can differentiate between healthy and cancerous cells or indicating early stages of the malignant phenotype. However, cfDNA extraction, sequencing, and analysis methods need further standardization to ensure reliable results. Detecting low-frequency cfDNA features could be challenging, due to the presence of background “noise” from non-tumor-derived DNA. cfDNA analysis can be expensive, limiting its widespread adoption in clinical practice, whereas the interpretation of cfDNA results can be complex, requiring advanced bioinformatics tools and expertise. The basic sequencing techniques (Sanger or pyrosequencing) need higher amounts of tumoral DNA to detect mutations, thus making them useful mainly in samples with a heavy tumor burden. Somatic mutations can be detected in cfDNA with satisfactory accuracy with PCR-based digital techniques (for example, ddPCR, NGS), which have additional costs and require additional training. Considering the average sensitivity for mutation detection in a sample (0.01%), it is important to understand that below that cut-off, there may be false-negative results.

Extracellular vesicles carry various molecular cargo, such as miRNAs, lncRNAs, and circRNAs. These cargos can be used as potential biomarkers for early HCC detection. EVs can be obtained non-invasively from blood and have a major advantage compared to other liquid biopsy techniques, as EVs protect their cargo from degradation, ensuring the stability of the biomarkers. Detecting low levels of tumor-derived EVs and their specific cargo components can be challenging due to the presence of background EVs from non-tumor cells, thus selection for specific tumor-derived EVs will be an important research goal. Methods for EV isolation, enrichment, and detection need further standardization to ensure accurate and reproducible results for clinical practice.

Combining EV-based biomarkers with other liquid biopsy methods or traditional serology biomarkers may enhance the overall diagnostic accuracy for early HCC detection, in the future.

It must be taken into account that detecting and validating liquid biopsy biomarkers for early HCC diagnosis is a difficult task. In the ideal setting, the studies must be performed in an HCC screening program, thus maximizing the chance for early diagnosis. Most of the existing studies were performed recruiting HCC cases diagnosed in various stages, including early HCC cases. Furthermore, the potential biomarkers should be subsequently validated prospectively, during HCC screening programs. It is possible that specific liquid biopsy biomarkers are positive even in the absence of a detectable tumor, by standard imaging methods, thus raising important ethical concerns. Nevertheless, these situations should point to the need for more aggressive surveillance to detect the cancer in the earliest stage possible, thus facilitating curative therapeutic procedures and patient survival.

As molecular biology technologies and AI-enhanced bioinformatics develop and continue to enter clinical practice, current limitations should be overcome, making liquid biopsy solutions useful tools to facilitate HCC diagnosis at an early stage.

In conclusion, it is still difficult to predict which liquid biopsy component or technology will find its way into routine clinical practice, as all existing molecular techniques analyzing liquid biopsy components have both strengths and limitations, emphasized by the present review. The specific screening method for early HCC detection should be cost-effective and acceptable for both patients and medical care systems. cfDNA fragmentation patterns for HCC detection show great promise to fit into this profile, as novel screening tool for early HCC detection. It is also possible that a combination of molecular and traditional serum biomarkers will, ultimately, be the best solution for HCC screening in the near future, as supported by multiple recent studies, if costs will also be affordable. The forthcoming years will, nevertheless, bring exciting new developments in the field of early HCC detection, using new liquid biopsy-based biomarkers.

## Author contributions

IM and RI have equally contributed to this work, and are sharing first authorship. IM, RI, GO, IP, and LG conceived the plan of the manuscript. IM, RI, SI, and RC performed the literature search. IM, RI, RC, SI, and SD have reviewed current literature and made the first draft of the paper. IM, RI, SI, and GO: Section 1. IM, RI, and SD: Section 2. IM, RI, and RC: Section 3. IM, RI, SI, SD, and GO: Section 4. GO, IP, and LG reviewed the manuscript. All authors contributed to the final version of the manuscript.

## Abbreviations

AFP, alpha-fetoprotein
BCLC, Barcelona Clinic Liver Cancer
bp, base pairs
CAZA, chromosome arm-level *z*-score analysis
cfDNA, cell-free DNA
CircRNA, circular RNA
CLD, chronic liver disease
CpG, 5’-C-phosphate-G-3’
CT, computer tomography
CTCs, circulating tumor cells
ctDNA, circulating tumor DNA
DCP, des-gamma-carboxy prothrombin
DELFI, DNA Evaluation of Fragments for Early Interception
DKK1, dikkopf-1
EASL, European Association for the Study of the Liver
ECOG, Eastern Cooperative Oncology Group
ELISA, enzyme-linked immuno-sorbent assay
EM-seq, enzymatic methyl sequencing
EVs, extracellular vesicles
GPC3, glypican-3
HBV, hepatitis B virus
HCC, hepatocellular carcinoma
HCV, hepatitis C virus
HIFI, 5-Hydroxymethylcytosine/ motIf/ Fragmentation/nucleosome footprint
LC, liver cirrhosis
lncRNA, long non-coding RNA
miRNAs, micro RNAs
MRI, magnetic resonance imaging
MVE, multivesicular endosome
MVs, microvesicles
NAFLD, non-alcoholic fatty liver disease
NASH, non-alcoholic steatohepatitis
NGS, next generation sequencing
OPN, osteopontin
PAGE-B, Platelet Age GEnder–HBV
PIVKA-II, protein induced by vitamin K absence-II
qRT-PCR, quantitative reverse-transcription PCR
TCGA, The Cancer Genome Atlas
TDS, targeted deep sequencing
TEPS, tumor-educated platelets
TGF-β1, tumor growth factor β1
US, ultrasonography
WES, whole exome sequencing
